# Patient-derived gastric cancer organoids for functional precision oncology: disease-specific bottlenecks and clinical readiness

**DOI:** 10.1016/j.tranon.2026.103065

**Published:** 2026-10-13

**Authors:** Chenyang Mi, Xuechao Liu, Xiaowen Tang, Guoliang Li

**Affiliations:** a Department of Radiation Oncology, The Affiliated Hospital of Qingdao University, Qingdao, Shandong, 266003, China; b Department of Medicinal Chemistry, School of Pharmacy, Qingdao University, Qingdao, 266071, China; c Department of General Surgery, The Affiliated Hospital of Qingdao University, Gastrointestinal Tumor Translational Medicine Research Institute of Qingdao University, Qingdao, Shandong, China

**Keywords:** Gastric cancer, Patient-derived organoids, Precision oncology, Drug sensitivity testing, Clinical readiness

## Abstract

Gastric cancer (GC) poses a distinct modeling challenge. The disease varies by region, histology, metastatic site, and treatment history. It also develops within a gastric niche shaped by acid, mucus, polarity, microbes, and stroma. Patient-derived organoids (PDOs) can preserve tumor-linked epithelial states and support living biobanks. They also enable genotype–phenotype studies and functional testing of therapeutic vulnerabilities. Their clinical value depends on representation, not model complexity alone. The assay must also return clear results within a useful clinical window.

This review evaluates GC PDOs as functional companion models for precision oncology rather than stand-alone treatment decision tools. We discuss four barriers that limit clinical readiness. Establishment bias reduces representation of diffuse-type, scirrhous, and signet-ring cell carcinomas. Standard organoid lumens do not reproduce gastric acidity, stratified mucus, or *Helicobacter pylori*-driven polarity loss. Long-term expansion can select culture-adapted clones and weaken clinical representativeness. PDO drug screening also remains difficult to align with urgent treatment timelines. Fresh-sample assays may shorten turnaround time but reduce scale and depth.

We further highlight biological insights gained from GC organoids. These include niche independence, epigenetic dependencies, ARID1A-linked vulnerabilities, and chemotherapy resistance. We propose a readiness framework based on six criteria: tumor authenticity, subtype coverage, niche fidelity, turnaround time, assay standards, and outcome validation. This framework may help define where GC PDOs can support clinical reasoning.

## Highlights

• GC PDO biobanks underrepresent diffuse and signet-ring tumors.
• Gastric niche mismatch limits infection and barrier modeling.
• Platform choice should match biological questions and clinical timelines.
• GC PDOs are companion models, not stand-alone decision tools.

## Graphical abstract

**Figure:**
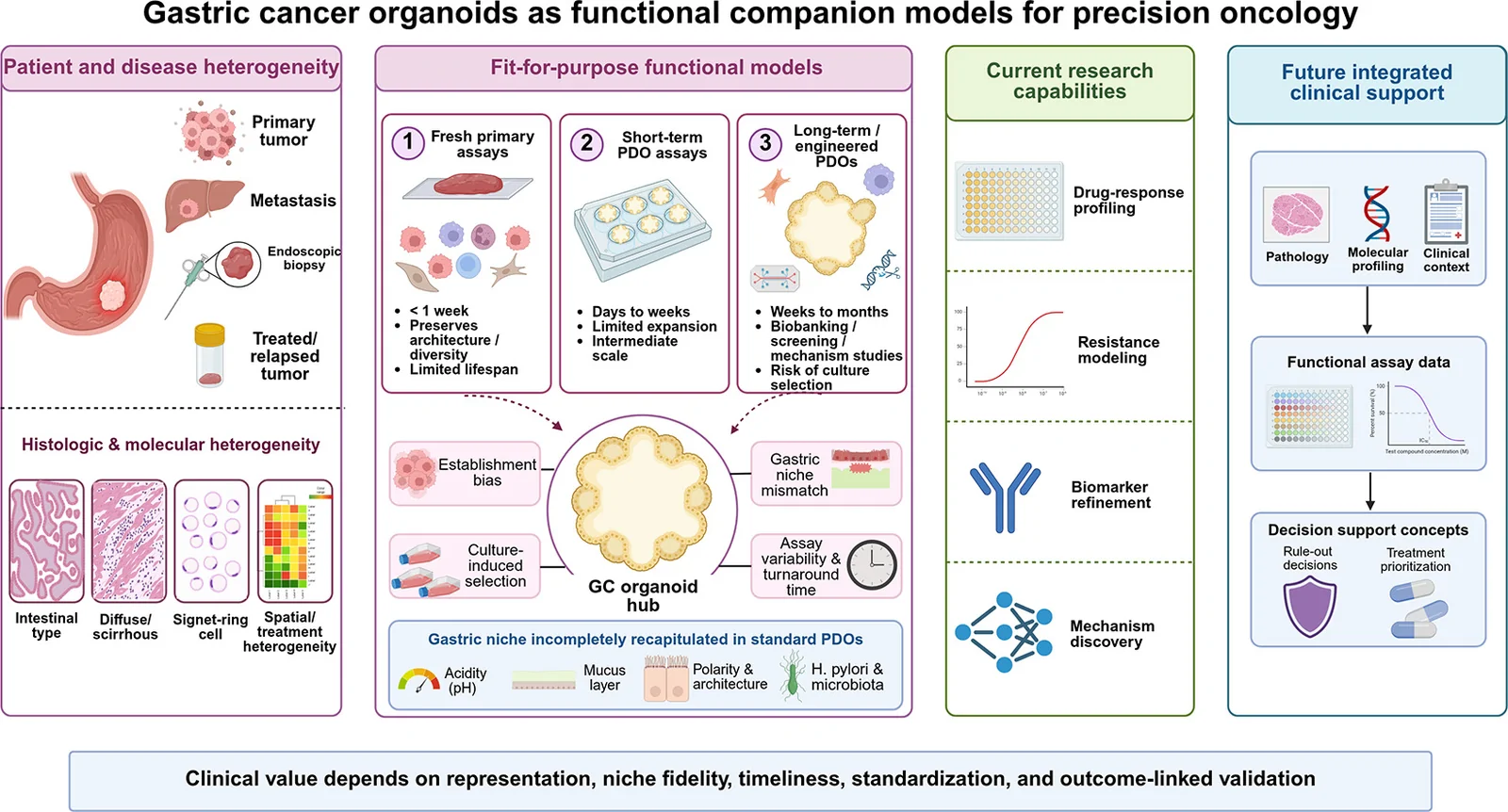

## Introduction: From organoid technology to functional precision oncology

GC remains a major cause of cancer morbidity and mortality [1]. The burden is especially high in East Asia and other high-incidence regions [1,2]. Yet the clinical challenge extends beyond late diagnosis and unequal treatment access. GC comprises diverse histological and molecular states [3]. These states differ in lineage programs, immune contexture, stromal dependence, metastatic behavior, and therapeutic vulnerability [4]. A single biopsy may therefore miss clinically relevant disease states. Static biomarker panels also provide an incomplete view [5]. Conventional monolayer cell lines lose much of the spatial and patient-linked context. This gap widens after therapy, when resistant subclones reshape the tumor population.

Patient-derived organoids (PDOs) can connect patient material to functional cancer modeling [6]. They support molecular profiling, perturbation studies, living biobanks, and drug response testing [6,7]. For GC, this value lies in measuring phenotype under controlled therapeutic pressure. PDOs may therefore complement static biomarker stratification in precision oncology. Yet this promise has led to an overly linear view. Some pipelines imply a smooth path from biopsy to organoid growth, screening, prediction, and treatment selection. Current evidence does not support that sequence as routine clinical practice [8,9]. GC PDOs are better viewed as functional companion models. They can support mechanism discovery, biomarker refinement, drug prioritization, and rule-out decisions.

This Review asks a focused question. Are GC PDOs ready to support functional precision oncology in clinical settings? We address this question through gastric-specific bottlenecks. These include under-representation of diffuse, scirrhous, and signet-ring phenotypes and mismatch with the native gastric lumen. They also include loss of stromal and immune niches, assay variability, and limited standardization [8]. A key practical issue is time. Long-term expandable PDOs offer scale and depth, but they may miss urgent treatment windows. Rapid fresh-sample assays may preserve native context, but they reduce experimental breadth. This framing moves the field beyond a catalog of technologies. A useful GC organoid model should be tumor-derived, subtype-representative, interpretable, reproducible, and clinically timely.

## What GC PDOs preserve, and what culture selects away

GC PDOs can preserve important features of the source tumor. Early GC organoid biobanks retained histological, genomic, and transcriptomic traits of patient tumors. These models also supported expansion, cryopreservation, molecular profiling, and drug testing [6,7,10]. This advance moved GC modeling beyond isolated cell lines. It created patient-linked resources for repeated functional studies.

GC PDOs occupy an intermediate position between highly reductionist models and systems that retain more native tissue context. Conventional two-dimensional cell lines are readily scalable but progressively lose patient-specific architecture and heterogeneity [9]. Multicellular spheroids provide simple three-dimensional organization, yet their biological complexity and long-term renewability are limited [11]. Tumor slices preserve native tissue architecture and microenvironmental components for short-term functional assays but are not readily expandable [12,13]. Patient-derived xenografts retain tumor features in vivo and support longitudinal studies, although they require longer timelines, greater resources, and host replacement of the human microenvironment [9]. In contrast, PDOs combine patient linkage, three-dimensional epithelial organization, renewable expansion, and experimental scalability, but incompletely preserve the native stromal and immune context [9,11]. These complementary strengths and limitations are summarized in Table 1.

**Table 1:** Comparative features of gastric cancer models for functional precision oncology.

**Model**	**Fidelity**	**Microenvironment**	**Throughput**	**Best use**
**2D cell lines**	Low-moderate	Minimal	High	Mechanistic studies; screening
**Spheroids**	Variable	Limited	Moderate	Short-term 3D phenotyping
**Tumor slices**	High	Native,short-lived	Low	Short-term pharmacodynamic testing
**PDXs**	High	Host-replaced	Low	In vivo validation
**PDOs**	High(epithelial)	Limited unless engineered	Moderate-high	Biobanking; perturbation and drug-response testing

**Note:** Ratings are qualitative and reflect relative model characteristics; performance may vary with specimen source, culture conditions, assay duration, and experimental purpose. PDXs, patient-derived xenografts; PDOs, patient-derived organoids.

Accordingly, model selection should be guided by the biological question and the required clinical time window rather than by model complexity alone. Robust growth does not prove disease representation. PDO establishment depends on many pre-analytical and biological variables [[14], [15], [16]]. These include ischemia time, transport conditions, contamination, dissociation method, viable tumor fraction, biopsy depth, stromal content, and medium composition. Each step can select cells that tolerate culture. Normal gastric epithelium may expand under supportive growth-factor conditions. It can outgrow malignant cells when tumor purity is low. This risk increases when authentication is incomplete. Tumor clones can also be selected in the opposite direction. Niche-independent, highly proliferative, or ECM-adapted clones may expand preferentially. Successful growth is therefore an operational milestone. It is not a validation endpoint.

Culture conditions also act as biological filters. Gastric epithelial organoids often require EGF, Noggin, R-spondin, Wnt activity, and pathway modulators [17]. Tumor clones may differ in their dependence on these niche factors. During gastric carcinogenesis, some clones acquire Wnt or R-spondin independence through distinct routes [18]. These patterns can reveal tumor biology. They can also alter clonal composition during culture. Passage number, freezing, thawing, matrix stiffness, and epithelial purification add further pressure. These steps may enrich states that grow well in vitro. Drug response can depend on minor clones, differentiation states, or microenvironmental support. Culture selection therefore has direct translational consequences.

Functional precision oncology requires layered validation. Identity control should confirm tumor origin and lesion linkage. Stability control should record passage number, genomic drift, and phenotype retention. Purity control should detect normal epithelial overgrowth [7,15,19]. Metadata should include sampling site, histology, prior treatment, tumor cellularity, culture formula, assay passage, sequencing data, and response metrics [16,19]. Without this traceability, a GC PDO biobank may lose clinical meaning. It may become a collection of expandable epithelial models rather than a representative patient-linked resource.

## Establishment bias across histopathological phenotypes

Establishment failure in GC PDO biobanks may be non-random and partly reflect tumor histopathology. Intestinal-, diffuse-, and mixed-type gastric cancers show distinct growth and architectural features, while diffuse and signet-ring phenotypes are characterized by poorly cohesive and infiltrative growth [[20], [21], [22]]. A recent meta-analysis of eight studies reported an overall pooled GC organoid establishment rate of 66.6%, with substantial between-study heterogeneity [14]. The pooled rate for signet-ring cell carcinoma was 32.7% [14]. In a small single-center cohort, organoids were established from 3/3 intestinal-type, 1/4 diffuse-type, and 2/2 mixed-type tumors [23]. These findings suggest potential histology-dependent establishment bias, although differences in cohort size, sample source, and culture conditions preclude direct comparison across studies. Representative findings are summarized in Table 2.

**Table 2:** Reported establishment outcomes across histopathological categories of gastric cancer patient-derived organoids.

Histopathological category	Establishment outcome	Evidence basis	Representation concern
Intestinal-type	3/3 [23]	Small single-center cohort	Higher than diffuse-type in the same cohort*
Diffuse-type	1/4 [23]	Small single-center cohort	Potential under-representation
Signet-ring cell carcinoma	32.7% (pooled) [14]	Meta-analysis	Potential under-representation
Mixed-type	2/2 [23]	Small single-center cohort	Insufficient evidence
Scirrhous-type gastric cancer	Successful establishment reported; rate not estimable [24]	Case-level evidence	Limited quantitative evidence

*Note: Histopathological categories reflect the terminology used in the cited studies and are not necessarily mutually exclusive. Establishment outcomes were reported under heterogeneous cohort sizes, sampling strategies, and culture conditions and should not be interpreted as direct cross-study comparisons. Based on a small single-center cohort.

Although intestinal-type tumors appear more compatible with current PDO culture systems in several small cohorts, this observation remains limited by heterogeneous sampling and culture conditions. Therefore, enrichment of intestinal-type phenotypes should be considered when interpreting PDO-based drug screening datasets.

Several biological features may contribute to this bias. Diffuse-type and signet-ring tumors often show poorly cohesive and infiltrative growth. E-cadherin dysfunction can weaken cell–cell adhesion and tissue architecture [25]. Conventional ECM dome culture favors epithelial clusters that readily form cystic or gland-like structures and may therefore disadvantage poorly cohesive tumor states. Scirrhous gastric cancer presents a distinct challenge because extensive desmoplasia and tumor–stroma interactions are central features of the disease [26]. Epithelial-only derivation may remove stromal signals that support tumor-cell survival and expansion. Biopsy-derived cultures add further constraints, including low tumor cellularity, normal epithelial contamination, and spatially restricted sampling [9,14].

These biases affect how PDO data should be interpreted. A high establishment rate does not ensure representative subtype coverage. Expandable PDO collections may preferentially retain culture-compatible tumor states, potentially biasing drug-response studies and biomarker discovery. Clinical studies may likewise be enriched for tumors that expand sufficiently for downstream testing [9,14]. Establishment failures should therefore be reported as part of platform performance rather than treated solely as technical loss. For drug-screening and precision-oncology studies, histological strata should be prespecified, and establishment attempts, failures, and successful cultures should be reported by subtype to avoid over-representation of culture-compatible tumors.

Subtype-aware strategies may help reduce this gap. Diffuse and signet-ring models may benefit from modified matrix composition or mechanics and tailored culture conditions [27]. Stromal support may be particularly relevant for scirrhous tumors [26]. Patient-matched fibroblasts and assembloid approaches can restore selected tumor–stroma interactions [28], whereas tissue-derived ECM may provide a more physiological matrix context [29]. These approaches should address defined representational limitations rather than add complexity for its own sake. The major establishment bottlenecks and resulting biases are summarized in Fig. 1.

**Fig. 1:**
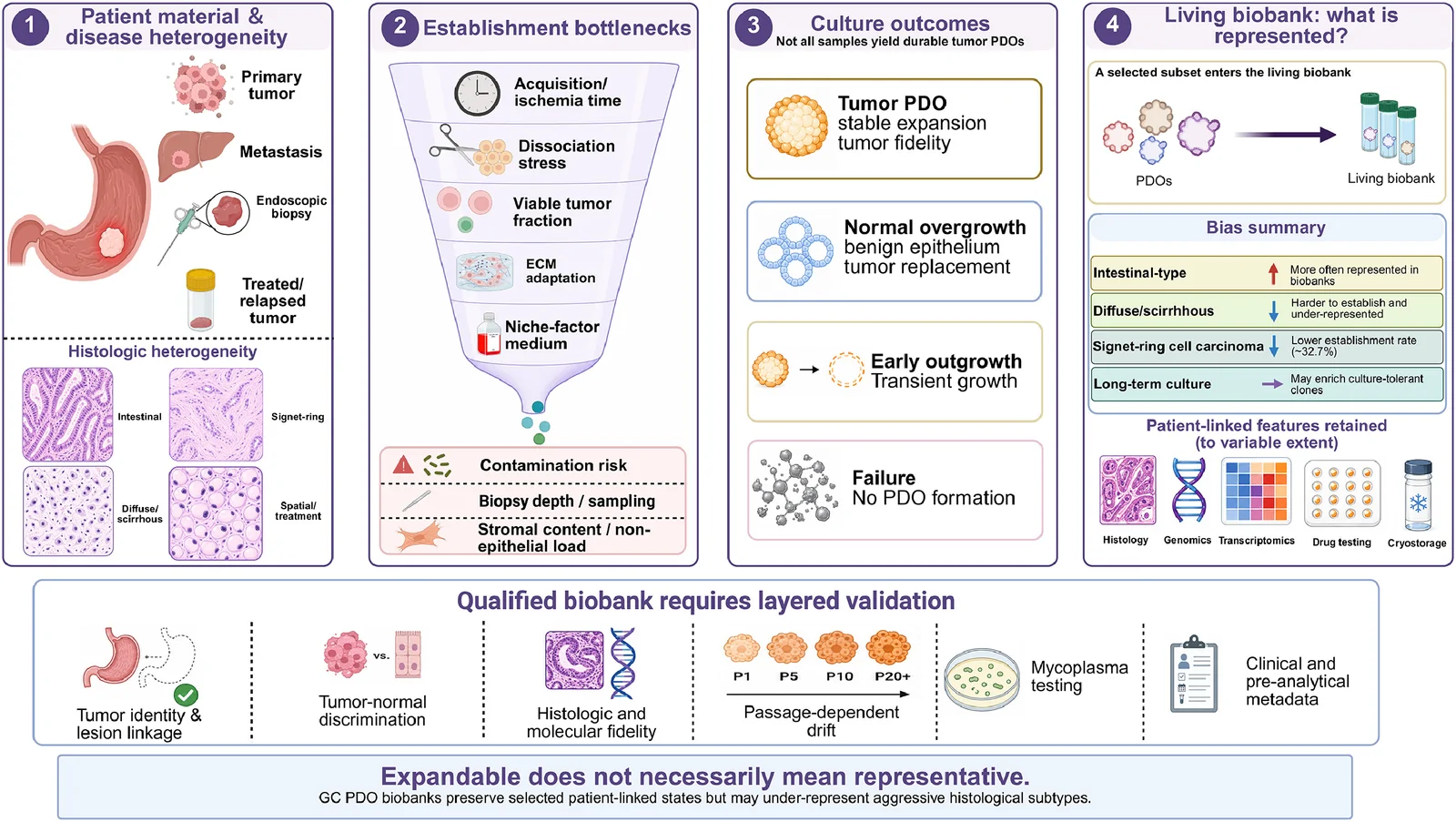
Establishment bottlenecks and representational bias in gastric cancer patient-derived organoid biobanks.

The schematic illustrates how biological and technical selection pressures influence the representativeness of gastric cancer patient-derived organoid (PDO) collections. Patient materials with diverse histological states, sampling conditions, and treatment histories undergo multiple culture-dependent selection steps. Establishment outcomes are shaped by factors including tissue quality, tumor cellularity, extracellular matrix adaptation, niche-factor dependence, stromal content, and contamination risk. These pressures may generate expandable PDOs, normal epithelial overgrowth, transient growth, or culture failure. Consequently, expandable biobanks may preferentially capture culture-adapted tumor states and under-represent diffuse-type, scirrhous, and signet-ring cell carcinomas. Layered validation integrating tumor identity, histological and molecular fidelity, passage stability, quality control, and clinical metadata is required to define model representativeness. Created in BioRender. mi, C. (2026) https://BioRender.com/xij3wv4

## Gastric niche mismatch: Acidity, mucus architecture, polarity, and H. pylori

The gastric lumen creates a distinct modeling problem. Standard 3D gastric organoids form a closed internal cavity. This cavity is not the native gastric lumen. The stomach contains local acid gradients, layered mucus, epithelial polarity, glandular structure, flow, and microbes [[30], [31], [32]]. These features regulate differentiation, barrier integrity, host–pathogen interaction, and drug exposure [31,32]. A GC PDO grown as an epithelial cyst may retain tumor-intrinsic programs. Yet it does not recreate the environment of chronic injury, inflammation, metaplasia, dysplasia, and malignant transformation. This mismatch limits how gastric physiology is interpreted.

Luminal pH is a major source of mismatch. Native gastric epithelium faces organized acid and mucus gradients [32]. Standard organoid lumens are enclosed and poorly controlled. Their internal pH may differ from the in vivo stomach [33]. This issue affects studies of acid injury, mucin barriers, *Helicobacter pylori* colonization, polarity, and luminal drug access [[32], [33], [34]]. It also weakens claims of gastric mucosal fidelity when pH is not measured. Several models can improve apical access. These include microinjection, apical-out organoids, air–liquid interface culture, and stomach-on-a-chip systems [[35], [36], [37]]. Luminal flow may also improve exposure control [31,37]. Each approach has trade-offs. Throughput, reproducibility, cell viability, and assay standardization may all be affected.

*H. pylori* shows why gastric organoids should provide mechanistic insight. They should not be cited only as culture milestones. Bartfeld and colleagues showed that human gastric epithelial organoids can be expanded. They also used these models to study bacterial infection [17]. Later work linked infection directly to epithelial architecture. CagA-positive *H. pylori* can target TP53BP2 (ASPP2) and disrupt the PAR polarity network, including PARD3 and MARK2, with associated mislocalization of SCRIB. These changes disrupt apical–basal polarity and favor bacterial colonization [38]. This mechanism links a gastric carcinogen to epithelial architecture and barrier dysfunction. More broadly, epithelial polarity determines whether luminal and basolateral compartments remain functionally distinct. Loss or inversion of polarity can alter apical accessibility, receptor localization, mucus secretion, microbial contact, and drug exposure. Polarity fidelity therefore affects how gastric organoid experiments on infection, barrier function, and luminal drug delivery should be interpreted. Models requiring direct apical access may benefit from polarity-controlled organoids or polarized gastric epithelial systems [35,36].

Mucus architecture is another missing feature. Gastric mucus controls bacterial access and protects epithelium from acid [32,39]. It also shapes diffusion and regional gastric identity [32]. Standard organoids may express mucin programs. Yet a cystic lumen does not reproduce a native mucus gel. It lacks physiological turnover and spatial layering [32,39]. This limitation may matter less for short-term cytotoxicity testing. It becomes critical for infection, acid injury, barrier failure, tumor–normal competition, and drug penetration. Future studies should define the required level of niche fidelity. Simple epithelial PDOs may be sufficient for intrinsic drug sensitivity. Models of infection, acid injury, mucus barriers, or apical exposure need stronger gastric niche control [30,31,34]. The major differences between the native gastric niche and standard epithelial PDOs, together with question-matched engineering strategies, are illustrated in Fig. 2.

**Fig. 2:**
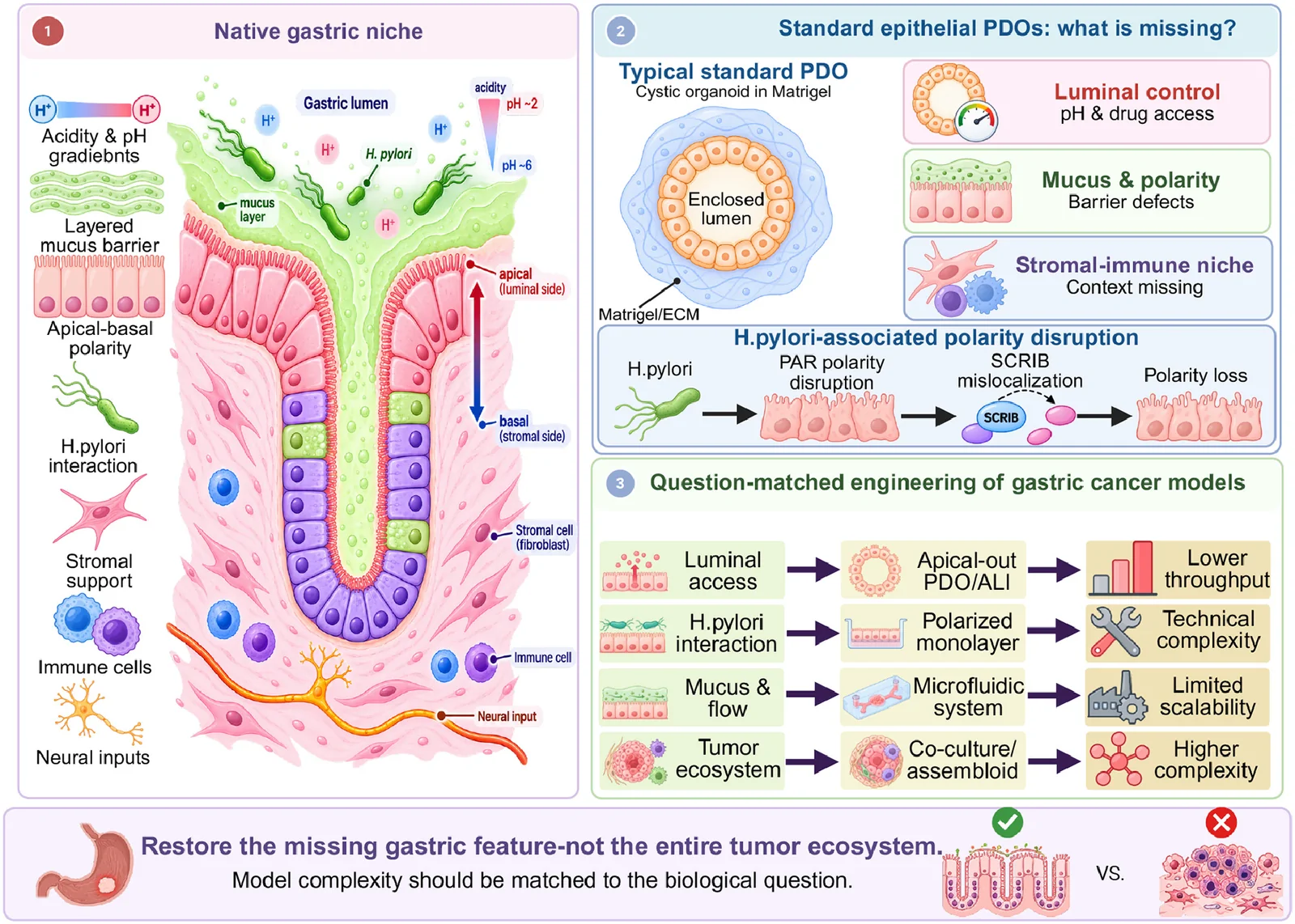
Gastric niche mismatch and question-guided engineering of gastric cancer patient-derived organoid models.

(A) The native gastric microenvironment comprises acidic luminal conditions, mucus architecture, epithelial polarity, glandular organization, microbial interactions, and stromal, immune, and neural components. (B) Conventional epithelial PDOs preserve tumor-intrinsic properties but incompletely reproduce gastric-specific features, including luminal control, apical access, mucus organization, and non-epithelial interactions. H. pylori-associated disruption of epithelial polarity is shown as an example of gastric niche-dependent biology. (C) Engineering strategies, including polarity control, apical exposure, luminal flow, matrix optimization, and selected stromal or immune integration, can restore specific biological features according to experimental requirements. Model complexity should therefore be determined by the biological question rather than by maximal ecosystem reconstruction. Created in BioRender. mi, C. (2026) https://BioRender.com/w3kyoox

## From technology catalog to biological discovery

Organoids are most useful when they reveal hidden disease mechanisms. Their value does not come from technical complexity alone. CRISPR, multi-omics, imaging, and computational models should not be listed as separate tools. They matter when they expose GC-specific dependencies, resistance states, or genotype–phenotype links. This section therefore focuses on discoveries, not methods.

Niche independence is a clear example. During gastric carcinogenesis, tumor clones can acquire Wnt or R-spondin independence through distinct routes [18]. This finding is more than a culture observation. It links genotype to growth-factor dependence, differentiation, and niche escape. Organoids provide a controlled system to test these changes. They also show a limitation of culture itself. The same conditions can reveal niche-independent states and enrich them over time.

Organoid engineering can also define context-specific vulnerabilities. In ARID1A-deficient human gastric organoids, CRISPR/Cas9 editing was combined with compound screening. This approach revealed selective sensitivity to BIRC5 (survivin) inhibition [40]. The finding has biological value because ARID1A alterations are clinically relevant in GC. Their effects may still depend on tissue context and lineage state. Gastric organoids allow these dependencies to be tested in human epithelial models. They reduce reliance on long-adapted cell lines.

CRISPR screening has extended this discovery framework. Negative-selection screening in GC PDOs identified an epigenetic vulnerability linked to the KDM1A–NDRG1 axis [41]. This result suggested a biomarker-guided strategy for KDM1A inhibition. Large-scale CRISPR screening in primary human 3D gastric organoids has also mapped gene–drug interactions [42]. These studies are important because they move beyond association. They use human gastric models to test causality.

Drug-pressure models add another layer of discovery. GC organoids selected under 5-FU pressure have revealed resistance-associated programs [43]. Oxaliplatin-resistant GC organoids have also linked resistance to myoferlin (MYOF)-dependent phenotypes [44]. Such models do not directly decide patient treatment. They help generate hypotheses for resensitization and resistance biomarkers. They also show how therapy can reshape functional tumor states.

Multidimensional readouts can strengthen these findings. Single-cell approaches help define lineage states, tumor microenvironment programs, and subtype-specific expression patterns in GC [4]. Spatial references further help evaluate whether organoid states match native gastric tissue organization [45]. High-content imaging can quantify organoid growth and morphology at scale [46]. Digitalized organoid pipelines can further improve segmentation and structural analysis [47]. Metabolic profiling may reveal cell-state plasticity that is not captured by growth curves alone [48]. Computational models trained on PDO data may connect in vitro phenotype with clinical response [49]. Yet these models need transparent metadata and multicenter validation [8,50]. Without this control, they may learn laboratory-specific culture effects. They may not capture robust gastric cancer biology. Representative biological discoveries enabled by GC organoids and the supporting multidimensional readouts are summarized in Fig. 3.

**Fig. 3:**
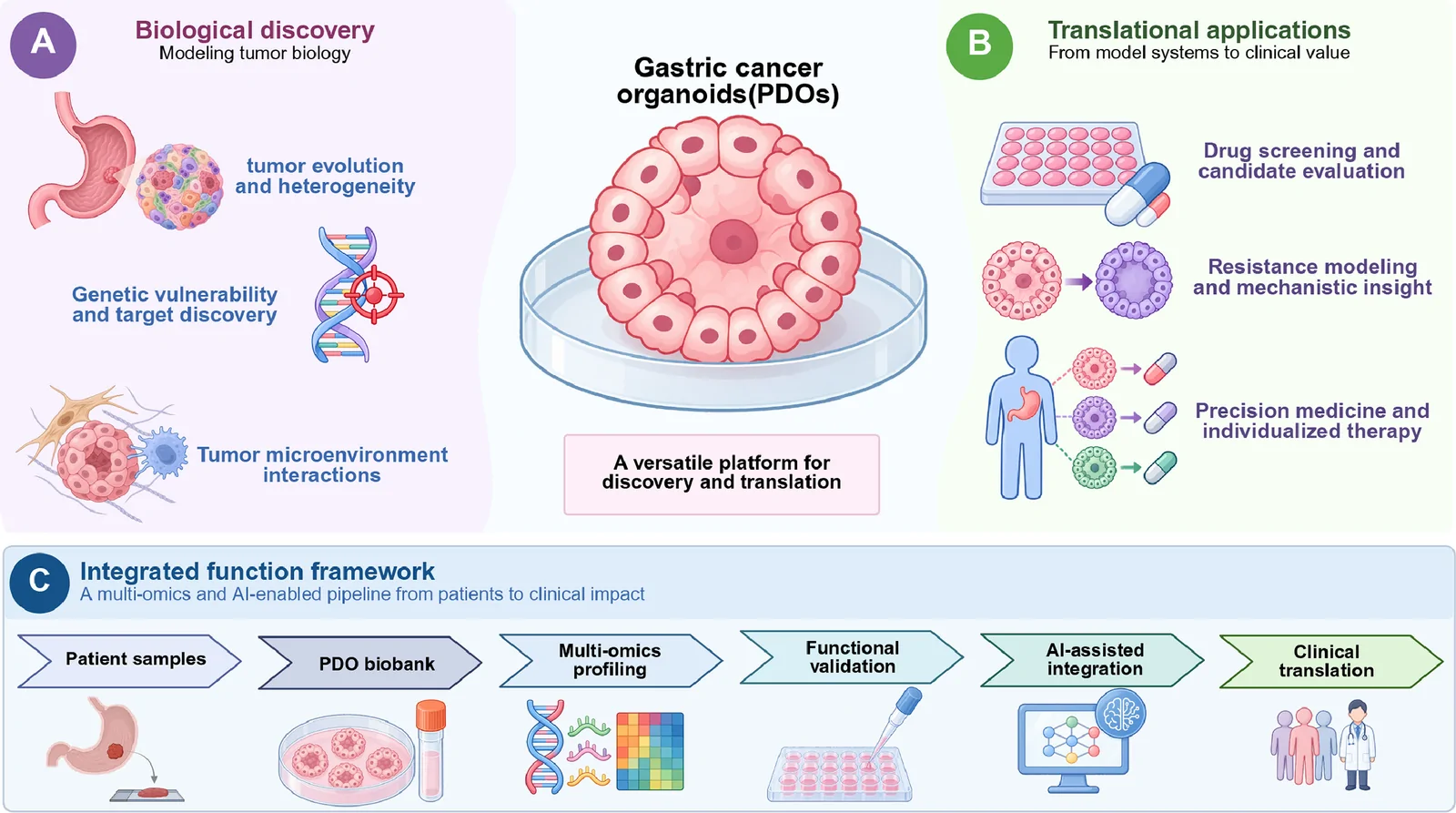
Biological discoveries enabled by gastric cancer organoid models.

(A) Gastric cancer organoids provide patient-relevant platforms for investigating tumor evolution and heterogeneity, genetic vulnerabilities and target discovery, and tumor–microenvironment interactions. (B) Translational applications include drug screening and candidate evaluation, resistance modeling and mechanistic investigation, and functional support for precision medicine. (C) The integrated framework illustrates a conceptual workflow linking patient samples, PDO biobanks, multi-omics profiling, functional validation, AI-assisted integration, and clinical translation. The graphical elements in panel C are conceptual and are not derived from newly generated experimental datasets. Created in BioRender. mi, C. (2026) https://BioRender.com/6i866wn

## Drug sensitivity testing: Promise, timelines, and platform trade-offs

Drug sensitivity testing is the most visible clinical use of GC PDOs. PDOs have tested fluoropyrimidines, platinum agents, taxanes, irinotecan, targeted agents, and combinations [7]. Several gastrointestinal studies also reported links between PDO response and patient outcome [51,52]. The main value is functional. The assay tests whether a patient-linked model responds to a drug under defined conditions. This evidence may help when genomic biomarkers are absent, unclear, or insufficient. It may also support treatment ranking when several regimens remain plausible.

Time remains the central translational barrier. Many clinical decisions must be made within days or weeks. Robust PDO establishment often takes longer. Expansion, quality control, and screening add further delay [53]. The difference between early outgrowth and useful expansion is therefore important. In the GC study by Zhao and colleagues, initial organoid lines were established in 57 of 73 cases. However, only 5 lines, corresponding to 6.8% of the enrolled cases, maintained sufficient proliferation for robust long-term expansion and DST. The remaining cultures ceased proliferation within a few passages. This distinction between early outgrowth and clinically usable expansion is critical, because a technically established PDO may still fail to deliver actionable results within the therapeutic decision window [7]. This gap shows a practical bottleneck. A platform can be scientifically successful but clinically late. Delayed results may still guide recurrence, later-line therapy, or resistance studies. They may not guide the first urgent treatment decision.

Long-term PDOs and fresh primary samples answer different questions. Long-term expanded PDO platforms may require weeks to months before screening results become available, whereas fresh tissue slices or minimally cultured cell suspensions can sometimes provide pharmacodynamic readouts within less than one week. Long-term PDOs support high-throughput screening and repeat testing [53]. They also enable molecular profiling, perturbation studies, and living biobanks. These models suit mechanism discovery, drug repurposing, and longitudinal research. They also fit settings where treatment decisions are less urgent. Their limits are clear. Establishment bias, clonal selection, and loss of native stroma can affect interpretation. Their turnaround time may also exceed the clinical window. Fresh platforms offer a different strength. Tissue slices and minimally cultured cell suspensions can preserve native architecture [12,13]. They may also retain clonal diversity and endogenous microenvironmental components. These assays can return results faster. Their limits include low cell yield, short lifespan, weak scalability, and limited repeat testing.

This trade-off argues against a universal PDO-DST pipeline. GC functional precision oncology should match the platform to the clinical question. Rapid fresh-sample assays may be more realistic for urgent first-line decisions. Short-term PDO assays may support treatment prioritization when options are similar. Long-term PDOs may be better for resistance evolution, recurrent disease, and biomarker discovery. Rule-out decisions also deserve attention. A model with strong negative predictive value may help exclude inactive regimens [52]. It does not need to act as a complete decision tool.

Response metrics shape clinical interpretation. Conventional IC50 values can be biased by different organoid growth rates [54]. GR metrics can reduce growth-rate bias in drug-response testing. NDR and NOGR further improve response quantification in cell-based and organoid assays [55,56]. These metrics still need full experimental context. Results should be linked to specimen source, passage number, medium, assay duration, drug exposure, endpoint, and clinical pharmacology [8]. GC adds further complexity because tumor purity and subtype composition vary widely. Reporting standards should include failed cultures and unsuccessful screens. Otherwise, clinical value may be overestimated from models that grow well enough for testing.

## Fit-for-purpose ecosystem engineering

Engineering should serve a defined biological question. It should not make every model more complex. Epithelial-only PDOs remain useful for tumor-intrinsic drug sensitivity. They can also model lineage programs and mutation-dependent vulnerabilities. Their limits become important in other settings. These include immune killing, stromal exclusion, desmoplasia, neural regulation, apical exposure, and diffusion gradients [57]. In these cases, selected ecosystem modules may improve relevance. Yet each added module brings variability and reduces throughput.

Immune-enabled organoid platforms can test defined immune interactions [58]. They allow studies of tumor-reactive T cells, antigen presentation, immune activation, and checkpoint-related perturbation [59,60]. This is relevant for GC. Immunotherapy response differs across MSI-high, EBV-positive, chromosomal instability, and genomically stable disease states. Biomarker-positive tumors also do not respond uniformly. Even so, immune co-cultures have clear limits. They often have short experimental windows and donor-dependent variation. Medium incompatibility can also affect tumor and immune cells. Immune composition may drift during culture. These models should therefore be viewed as functional immune assays. They are not complete reconstructions of the tumor immune microenvironment.

Stromal modules are especially important for diffuse and scirrhous GC [26]. Patient-matched fibroblasts, tissue-derived matrices, and assembloids can model matrix remodeling and invasion [28,29]. They can also capture epithelial–mesenchymal plasticity and stroma-mediated drug tolerance. These systems may help address establishment bias. They can provide survival cues for tumors that fail in epithelial-only culture. This benefit requires careful tracking. Stromal co-culture can obscure tumor-specific readouts. Tumor and stromal compartments should therefore be measured separately.

Microfluidic, matrix-engineered, and bioprinted systems address exposure and tissue geometry [37,61]. They do more than add extra cell types. Stomach-on-a-chip platforms can impose luminal flow and mechanical cues [37]. Defined matrices and tissue-derived ECM can reduce Matrigel dependence [29]. Bioprinting may improve geometry control and automated testing [61,62]. These systems should be used when they address a specific GC limitation. Relevant examples include luminal exposure, matrix dependence, and stromal architecture. Vascular or perfusion modules need more caution. They should not be emphasized without disease-specific evidence. They also need a clear experimental purpose. For clinical readiness, model selection matters more than model complexity.

## A clinical-readiness framework for GC PDOs

GC PDO research now needs a readiness framework. Another inventory of platforms would add limited value. We propose six linked criteria. Tumor authenticity should be verified by molecular or histological evidence. Each culture should also be linked to the sampled lesion [15]. Subtype representativeness should be reported with success and failure rates [14]. This is especially important for diffuse-type, scirrhous, and signet-ring carcinomas. Niche fidelity should match the biological question [15]. Some studies require gastric acidity, mucus architecture, epithelial polarity, or *H. pylori* exposure. Others require immune cells, stromal modules, flow, or apical access. Turnaround time should fit the intended clinical use [63]. Assay design also needs predefined standards [8]. These include response metrics, passage number, medium, matrix, seeding density, endpoint, and drug exposure. Outcome-linked validation remains essential. Organoid-guided interpretation should be tested in defined clinical settings [8,52]. The platform–time–purpose framework and the six clinical-readiness criteria for GC PDOs are integrated in Fig. 4.

**Fig. 4:**
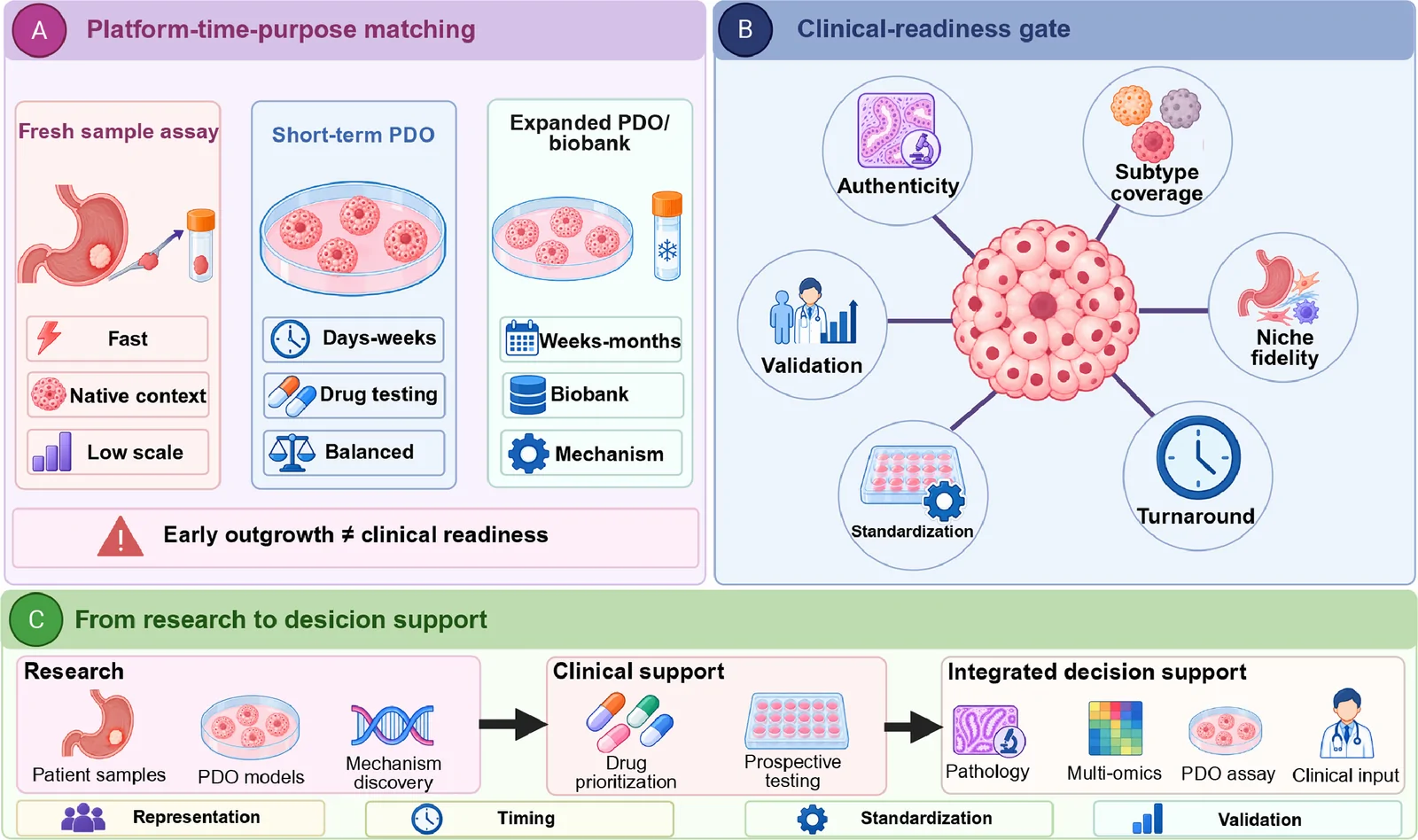
Platform–time–purpose matching and clinical-readiness framework for gastric cancer patient-derived organoids.

(A) Different PDO-based platforms provide distinct advantages depending on biological context and clinical requirements. Fresh primary-sample assays may preserve native tumor features and provide rapid functional information but have limited scalability. Short-term PDO assays offer intermediate flexibility, whereas long-term expanded PDOs support repeated testing, biobanking, and mechanistic investigations but require longer preparation and may experience culture selection. (B) Clinical readiness of gastric cancer PDOs should be evaluated across six interconnected dimensions: tumor authenticity, subtype coverage, niche fidelity, turnaround time, assay standardization, and outcome-linked validation. (C) The proposed framework separates current research applications from investigational clinical-support use and future integrated decision-support models. Gastric cancer PDOs should function as complementary tools that integrate with pathology, molecular profiling, and clinical information rather than independent treatment-selection systems. Created in BioRender. mi, C. (2026) https://BioRender.com/2sbai2s

This framework separates current capability from future clinical vision. Current capabilities include patient-linked epithelial models and living biobanks. They also include perturbation studies, drug-response assays, and selected ecosystem models. The main bottlenecks are representation, timing, standardization, and validation [63]. The future vision should not be an automated treatment-selection pipeline [9]. GC PDOs should not independently choose therapy. A more realistic goal is integrated decision support [64]. In this model, molecular profiling, pathology, clinical status, and functional assays inform treatment reasoning together.

Prospective validation should follow the intended use [8]. A rule-out assay needs different endpoints from a second-line prioritization study [52]. A resistance-discovery study also requires a different design. Failed cultures and delayed assays should be reported [14,63]. Discordant predictions and negative results should also be included. These data define the limits of clinical utility. Ethical and governance issues are part of readiness as well. Clinically linked biobanks require consent, traceability, and data protection [50,65]. They also need rules for redistribution. Investigators should clearly explain the status of organoid results. Patients and clinicians should know whether results are research findings or actionable evidence [65].

## Conclusions

GC PDOs have moved beyond niche-dependent epithelial cultures. They now support functional studies of tumor heterogeneity, drug vulnerability, and resistance. Their value does not lie in recreating the full tumor ecosystem. It lies in testing human tumor material under defined conditions. For functional precision oncology, the key question has changed. Researchers should ask which tumors are represented. They should also ask which gastric niche features are missing. Turnaround time and clinical validation are equally important.

The field should move from technology enthusiasm to model-aware readiness. Establishment bias against diffuse, scirrhous, and signet-ring tumors needs careful attention. This bias is both biological and clinical. Gastric niche mismatch also affects interpretation. Acid exposure, mucus architecture, epithelial polarity, and *H. pylori* interactions shape disease mechanisms. Drug sensitivity testing should be judged against clinical timelines. Platform trade-offs should also be reported clearly. Ecosystem engineering should serve a defined purpose. It should not add complexity for its own sake.

GC PDOs may become reliable functional companion models if these principles are followed. They can complement molecular profiling and refine treatment reasoning. They may also help identify resistance mechanisms and exclude weak treatment options. They should not be presented as stand-alone clinical decision tools. Their clinical value will depend on representation, timing, standardization, and outcome-linked validation.

## Funding

This work was supported by the National Natural Science Foundation of China (No. 82403565), the Shandong Provincial Natural Science Foundation (ZR2023QH276 and ZR2024QH448), and the Open Project Program of Shandong Key Laboratory of Pathogenesis and Prevention of Brain Diseases.

## Data availability

No new datasets were generated or analyzed in this review. Data sharing is not applicable to this article.

## CRediT authorship contribution statement

**Chenyang Mi:** Writing – original draft, Visualization, Investigation, Conceptualization. **Xuechao Liu:** Writing – review & editing, Validation. **Xiaowen Tang:** Writing – review & editing, Supervision, Funding acquisition, Conceptualization. **Guoliang Li:** Writing – review & editing, Supervision, Funding acquisition, Conceptualization.

## Declaration of competing interest

The authors declare that they have no known competing financial interests or personal relationships that could have appeared to influence the work reported in this paper.
